# Distinct Roles for Intra- and Extracellular Siderophores during Aspergillus fumigatus Infection

**DOI:** 10.1371/journal.ppat.0030128

**Published:** 2007-09-07

**Authors:** Markus Schrettl, Elaine Bignell, Claudia Kragl, Yasmin Sabiha, Omar Loss, Martin Eisendle, Anja Wallner, Herbert N Arst, Ken Haynes, Hubertus Haas

**Affiliations:** 1 Biocenter-Divison of Molecular Biology, Innsbruck Medical University, Innsbruck, Austria; 2 Department of Molecular Microbiology and Infection, Imperial College London, London, United Kingdom; Johns Hopkins University, United States of America

## Abstract

Siderophore biosynthesis by the highly lethal mould Aspergillus fumigatus is essential for virulence, but non-existent in humans, presenting a rare opportunity to strategize therapeutically against this pathogen. We have previously demonstrated that A. fumigatus excretes fusarinine C and triacetylfusarinine C to capture extracellular iron, and uses ferricrocin for hyphal iron storage. Here, we delineate pathways of intra- and extracellular siderophore biosynthesis and show that A. fumigatus synthesizes a developmentally regulated fourth siderophore, termed hydroxyferricrocin, employed for conidial iron storage. By inactivation of the nonribosomal peptide synthetase SidC, we demonstrate that the intracellular siderophores are required for germ tube formation, asexual sporulation, resistance to oxidative stress, catalase A activity, and virulence. Restoration of the conidial hydroxyferricrocin content partially rescues the virulence of the apathogenic siderophore null mutant *ΔsidA*, demonstrating an important role for the conidial siderophore during initiation of infection. Abrogation of extracellular siderophore biosynthesis following inactivation of the acyl transferase SidF or the nonribosomal peptide synthetase SidD leads to complete dependence upon reductive iron assimilation for growth under iron-limiting conditions, partial sensitivity to oxidative stress, and significantly reduced virulence, despite normal germ tube formation. Our findings reveal distinct cellular and disease-related roles for intra- and extracellular siderophores during mammalian *Aspergillus* infection.

## Author Summary

Patients with suppressed immune systems due to cancer treatments, HIV/AIDS, organ transplantation, or genetic disorders are at high risk of infection with the ubiquitously present fungal pathogen Aspergillus fumigatus. Treatments for this disease, collectively termed invasive aspergillosis, are often not successful, and prospects for survival can be slim. A. fumigatus produces small molecules, termed siderophores, for acquisition and storage of iron, an element essential for growth. We found that these siderophores are crucial for virulence of A. fumigatus because their removal (by gene deletion) prevents or lessens disease in a mouse model of invasive aspergillosis. Siderophores are not produced by humans so they present good prospects for new therapies, as drugs that specifically target siderophore production, rather than activities shared by humans and fungi, are less likely to affect patients adversely.

## Introduction

Animals strategically withhold iron during infection to combat invading microbes [1,2]. Consequently, the ability to obtain iron from the host, both for essential metabolism and to cope with reactive oxygen species produced by phagocytic cells [3], is a feature of most pathogens. The intimate coupling of iron uptake and storage with resistance to oxidative stress requires that all organisms strike a fine balance between the two; for example, catalases and peroxidases need heme as a cofactor [4], but iron overload or incorrect storage can result in, or exacerbate, oxidative stress via Haber–Weiss/Fenton chemistry [5].

Aspergillus fumigatus is a saprophytic mould that has become the most common airborne fungal pathogen to cause disease in humans. Global ubiquity, and the infectious cycle of this pathogen, is perpetuated by prolific production of asexual spores termed conidia. Conidial germination in the human lung, following spore inhalation, represents the initiating event of pulmonary disease. Three important steps can be distinguished during spore germination: activation of the resting spore to appropriate environmental conditions, isotropic growth that involves water uptake and wall growth (termed swelling), and polarized growth that results in the formation of a germ tube from which the new mycelium originates [6,7].

A. fumigatus causes a spectrum of diseases, depending upon the status of the host. Individuals with pre-existing structural lung disease, atopy, occupational exposure, or impaired immunity are susceptible to infection [8]. Invasive aspergillosis is now the most common cause of death due to fungal infection, occurring in up to one-quarter of transplant recipients or patients undergoing therapy for haematological malignancies, and 3% of AIDS patients. Typically, mortality associated with this disease reaches 50%–100%, due to difficulties with diagnosis and treatment [9]. Allergic bronchopulmonary aspergillosis is an A. fumigatus–induced respiratory disease usually found in atopic individuals [10] that can be life threatening, and frequently occurs in patients suffering from bronchial asthma, bronchiectasis, or cystic fibrosis [11].

A. fumigatus lacks specific uptake systems for host iron sources such as heme, ferritin, or transferrin. However, it employs two high-affinity iron uptake systems, reductive iron assimilation (RIA) and siderophore-assisted iron uptake, both of which are induced upon iron starvation [12]. RIA involves reduction of ferric to ferrous iron and subsequent uptake of ferrous iron by the FtrA/FetC complex, an activity that is blockable with the ferrous iron-specific chelator bathophenanthroline disulfonate (BPS) [12]. Siderophores are low molecular mass, ferric iron–specific chelators [13,14]. Like its mildly pathogenic relative *Aspergillus nidulans, A. fumigatus* produces three hydroxamate-type siderophores: extracellular fusarinine C (FSC) and triacetylfusarinine C (TAFC), and intracellular ferricrocin (FC) [12,15].

**Figure 1:**
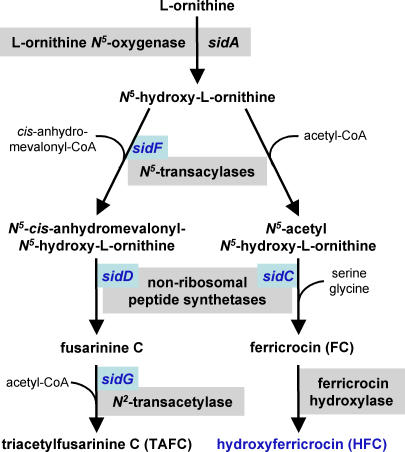
Postulated Siderophore Biosynthetic Pathway of A. fumigatus Steps identified during this study are in blue.

FSC is an *N^2^-*acetyl-lacking precursor of TAFC, a cyclic tripeptide consisting of three *N^2^-*acetyl-*N^5^*-*cis*-anhydromevalonyl-*N^5^*-hydroxyornithine residues linked by ester bonds. FC is a cyclic hexapeptide with the structure Gly-Ser-Gly-(*N^5^-*acetyl-*N^5^*-hydroxyornithine)_3_ [13]. Once secreted, FSC and TAFC mobilize extracellular iron for subsequent uptake [16], and in *A. nidulans,* FC is involved in intracellular conidial and hyphal iron storage [17]. The postulated biosynthetic pathway according to Plattner and Diekmann [18] is shown in Figure 1. The initial biosynthetic step, shared by pathways of both intra- and extracellular siderophore biosynthesis, is catalyzed by the ornithine-*N^5^*-monooxygenase SidA, the mutational inactivation of which abolishes siderophore biosynthesis, completely attenuating virulence in neutropenic mice and demonstrating the importance of the siderophore system in establishing infection [12]. Apart from A. nidulans SidC, the nonribosomal peptide synthetase essential for FC synthesis, other components of *Aspergillus* siderophore biosynthetic pathways have remained uncharacterized at the molecular level. In this study we identified the A. fumigatus FC synthetase SidC, and the TAFC biosynthetic enzymes SidD, SidF, and SidG. Moreover, we describe a novel A. fumigatus siderophore, hydroxyferricrocin (HFC), employed for conidial iron storage. Phenotypic characterization of respective mutant strains reveals distinct roles for extracellular and intracellular siderophores in iron metabolism, resistance to oxidative stress, and virulence.

## Results

### Identification and Functional Analysis of *A. fumigatus* Siderophore Biosynthetic Genes

**Figure 2:**
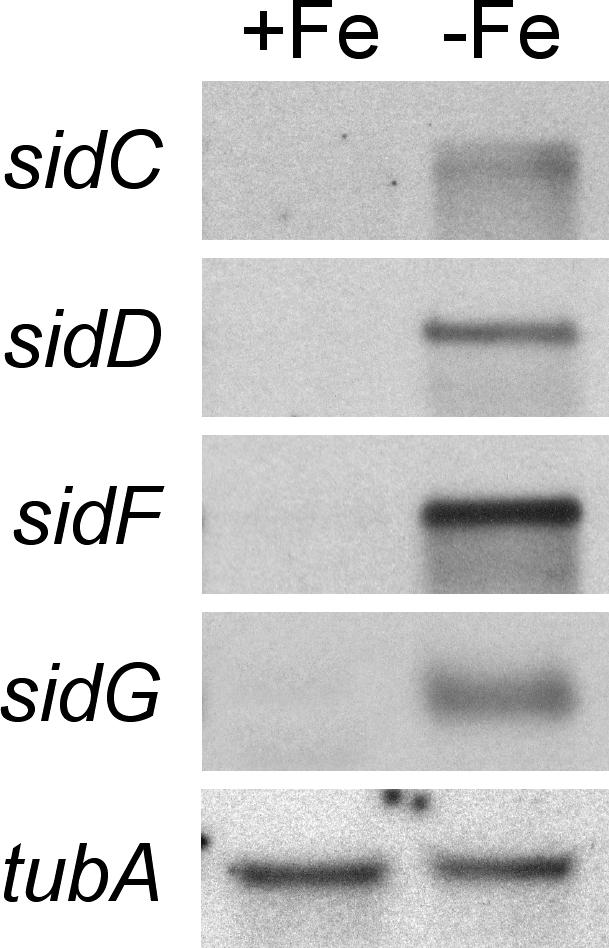
Northern Analysis of *A. fumigatus sidC, sidD, sidF,* and *sidG* Following growth for 24 h during iron starvation (−Fe) and sufficient iron (+Fe), total RNA was isolated from A. fumigatus ATCC46645. As a loading control, blots were hybridized with the β-tubulin encoding *tubA* gene of A. fumigatus.

Most ascomycetes and basidiomycetes produce siderophores, but well-known exceptions are Saccharomyces spp., Candida spp., and Cryptococcus spp. [19]. As a first step in our analyses, we screened the recently released A. fumigatus genomic sequence [20] for genes putatively involved in siderophore biosynthesis. We expected such genes to be conserved among siderophore-producing organisms, including fungi and bacteria, and to be upregulated by iron starvation. Northern analysis identified four candidate genes in A. fumigatus, termed *sidC* (Afu1g17200), *sidD* (Afu3g03420), *sidF* (Afu3g03400), and *sidG* (Afu3g03650) (Figure 2)*.* SidC and SidD are nonribosomal peptide synthetases involved in biosynthesis of FC and TAFC, respectively. SidC displays high identity (55%) to A. nidulans SidC [21], and is conserved in most siderophore-producing fungi. SidD is conserved among siderophore-producing ascomycetes and is subject to iron-mediated transcriptional repression [22]. With respect to its two-module structure and amino acid sequence, SidD displays high similarity to NPS6, which was recently shown to be involved in biosynthesis of TAFC- and coprogen-type siderophores in the plant pathogens *Cochliobolus heterostrophus, Cochliobolus miyabeanus, Alternaria brassicicola,* and *Fusarium graminearum* [23]. SidF homologs can be found among hydroxamate-producing fungi and numerous bacterial species; for example, the Escherichia coli homolog IucB, which displays 38% identity, is an *N^5^*-hydroxylysine:acetyl coenzyme A–*N^5^*-transacetylase that is essential for synthesis of the siderophore aerobactin [24]. SidG homologs, which belong to the *N*-acetyltransferase family (GNAT, pfam00583.11), are known to exist thus far in only two other fungal species, A. nidulans and *Giberella zeae,* but are present in several bacteria, such as *Caulobacter crescentus, Desulfovibrio vulgaris, Streptomyces coelicolor,* and Pseudomonas aeruginosa (http://www.ncbi.nlm.nih.gov/BLAST/).

**Figure 3:**
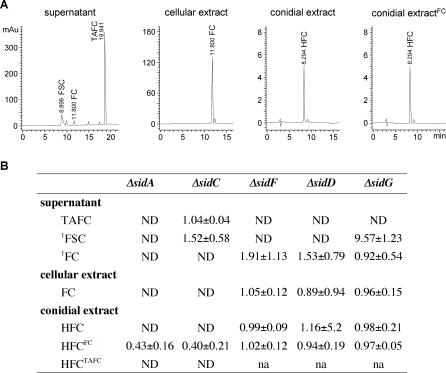
Extra- and Intracellular Siderophore Production of *A. fumigatus wt, ΔsidA, ΔsidC, ΔsidD, ΔsidF,* and *ΔsidG* (A) Representative HPLC analysis of culture supernatants, cell extracts, and conidial extracts of the *wt*. Units are given in milli absorption units (mAu). (B) Quantification of siderophore production of *ΔsidA, ΔsidC, ΔsidD, ΔsidF,* and *ΔsidG* normalized to that of the *wt* after 24 h of growth in iron-depleted conditions. For HPLC analysis of the conidial siderophore, the *wt* and mutant strains were grown for 5 d either with 0.5 mM FeSO_4_, 10 μM FC (^FC^), or 10 μM TAFC (^TAFC^), respectively, as iron source. ^1^The FSC and FC contents in *wt* supernatant represented 12.0±3.1% and 1.5±3.1% of the TAFC content, respectively. The data represent the means ± standard deviations of results from three independent experiments. ND, not detected; na, not analyzed.

To analyze the respective functions of SidC, SidD, SidF, and SidG, we generated gene deletion mutants in A. fumigatus ATCC46645 (*wt*) by replacement with the hygromycin B resistance (*hph*) marker termed *ΔsidC, ΔsidD, ΔsidF, and ΔsidG,* respectively*.* To assess the impact of gene deletion on siderophore biosynthesis in each mutant, background siderophore production was quantified using high-performance liquid chromatography (HPLC) analysis of both culture supernatants and cell extracts following growth for 24 h under siderophore-derepresssing (see Figure 2) iron-depleted conditions (Figure 3) and compared to that of the *wt* and of the ornithine-*N^5^*-monooxygenase mutant, *ΔsidA*, which lacks both intra- and extracellular siderophores. The *wt* accumulated 7 mg FC per gram dry weight of mycelium, and excreted 42 mg TAFC per gram dry weight. The supernatant also contained the direct TAFC precursor FSC, approximating 12% of the mycelial TAFC content, and FC was detectable in trace amounts (Figure 3A). SidC deletion abolished FC synthesis as measured from hyphal extracts but had no influence on TAFC and FSC production. Conversely, deletion of *sidD* or *sidF* prevented synthesis of TAFC and FSC without affecting FC production. SidG deletion eliminated TAFC production but increased FSC production about 10-fold, that is, the FSC reached in amount about the TAFC content of the *wt.* FC accumulation was not affected in *ΔsidG.*

Taken together, the siderophore production pattern of the deletion mutants, the features of the gene products, and the predicted siderophore biosynthetic pathway [18] strongly suggest that (i) *sidC* encodes the nonribosomal peptide synthetase involved in ferricrocin biosynthesis, (ii) *sidD* encodes the nonribosomal peptide synthetase responsible for TAFC biosynthesis, (iii) *sidF* encodes *N^5^*-hydroxyornithine:*cis* anhydromevalonyl coenzyme A–*N^5^*-transacylase, and (iv) *sidG* encodes FSC–acetyl coenzyme A–*N^2^*-transacetylase (Figure 1). For each deletion mutant, three independent complemented strains were subjected to siderophore and phenotypic analysis. Complementation (see Materials and Methods) reversed all mutant phenotypes (Figure S1), definitively linking the phenotypes to inactivation of the respective gene.

### A. fumigatus Employs a Novel Siderophore, HFC, as a Conidial Iron Storage Compound

*A. nidulans, Aspergillus ochraceus,* and Neurospora crassa employ FC as both a hyphal and a conidial iron storage compound [21,25]. In stark contrast, HPLC analysis of *A. fumigatus wt* conidial extract revealed a lack of FC. Instead, we were able to detect the presence of a structurally distinct siderophore indicated by a different retention time (Figure 3A). Its synthesis requires SidA and SidC but not SidD, SidF, or SidG, as indicated by conidial siderophore analysis of the respective deletion mutants (Figure 3B). When the spores were generated on medium supplemented with FC, but not TAFC, *ΔsidA* and *ΔsidC* conidia contained this novel siderophore, suggesting that it is derived from FC. TAFC addition did not lead to HFC formation (Figure 3B), negating the possibility that FC-bound iron induces HFC formation. Consistently, high-resolution mass spectrometry of this compound gave two molecular masses m/z (M+H)^+^ 787.2432 matching C_28_H_45_N_9_O_14_Fe (calculated molecular mass 787.2435), and (M+Na)^+^ 809.2253 matching C_28_H_44_N_9_O_14_FeNa (calculated molecular mass 809.2255) (unpublished data), suggesting that the conidial siderophore of A. fumigatus is derived from ferricrocin by hydroxylation; therefore, we termed it hydroxyferricrocin (HFC). Importantly, these data also show that conidia of *ΔsidA* and *ΔsidC* strains, which lack all intracellular siderophores, can be loaded with HFC by supplementation with FC during sporulation.

Notably, the total iron content of HFC-deficient conidia of both *ΔsidA* and *ΔsidC* strains was reduced about 67% compared to that of *wt* (Table 1). Conidial loading of either mutant with HFC by supplementation of the sporulation medium with the HFC precursor FC largely reconstituted the *wt* iron content (Table 1), demonstrating that HFC represents a major A. fumigatus conidial iron storage compound. All aspects of deletion-associated defects described above are completely rescued by gene-mediated complementation (Figure S1), directly linking observed phenotypic traits to deletion of the relevant siderophore biosynthetic pathway genes.

**Table 1:**
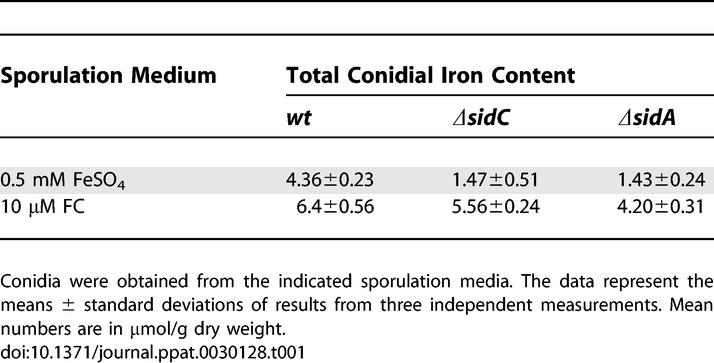
Iron Content of *A. fumigatus wt, ΔsidC,* and *ΔsidA* Conidia from Different Sporulation Media

**Figure 4:**
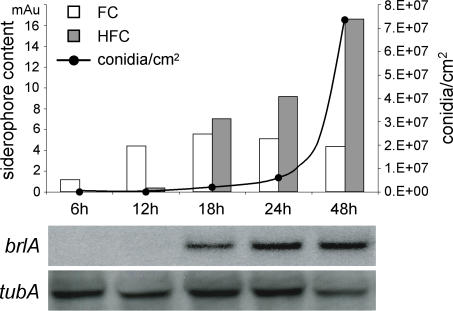
Time Course Analysis of Siderophore Content and of *brlA* Expression during Conidiation *A. fumigatus wt* cells were grown for 24 h under iron-replete liquid culture and subsequently transferred to iron-replete solid media. After growth on solid media up to the indicated time points, FC and HFC content was analyzed, as well as *brlA* expression. As a loading control, *tubA* was used. Quantity units of siderophore determination are given in milli absorption units (mAu).

As HFC was detected neither in the supernatant nor in hyphae of *wt* liquid cultures (Figure 3), we analyzed its potential developmental regulation by assessing siderophore content, conidia production, and expression of *brlA*, which encodes an early conidiation-specific transcription factor [26,27], over a time course spanning conidiation. HFC synthesis started concomitantly with *brlA* expression (Figure 4) and paralleled conidia production, indicating that its synthesis is developmentally regulated.

**Figure 5:**
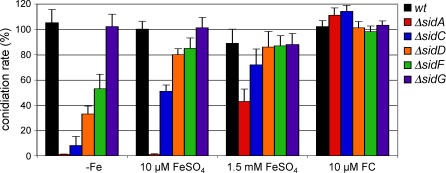
A. fumigatus Conidiation Rates in *wt* and Siderophore Biosynthetic Mutant Backgrounds 10^6^ conidia of fungal strains were point inoculated in the center of minimal medium plates containing the indicated iron source. Conidia produced by 1 cm^2^ were counted after 120 h of incubation at 37 °C. The *wt* conidia count was 4.5 × 10^8^. The data represent the means ± standard deviations of results from three independent experiments.

We have previously shown that SidA deficiency causes absence of asexual sporulation during iron-depleted conditions partially curable by increased iron availability and completely restored by FC supplementation [12]. *ΔsidC* displayed a similar conidiation phenotype as *ΔsidA* (Figure 5), but *ΔsidC* produced more conidia than *ΔsidA* at the same iron concentration. Hence, FC or HFC appears to be specifically important for optimal sporulation. *ΔsidD* and *ΔsidF* showed decreased conidiation only during iron-depleted conditions, suggesting that this defect is caused by iron deficiency due to lack of siderophore-mediated iron uptake. *ΔsidG* displayed a *wt* conidiation rate. In view of sporulation defects in siderophore mutant backgrounds, generation of spores from all strains for which phenotypic and virulence testing is described was performed on iron-supplemented medium unless otherwise stated.

### HFC Is Required for Germ Tube Formation during Iron-Depleted Conditions

The importance of FC for efficient germ tube formation from A. nidulans conidia, where it maintains the conidial siderophore, has been previously demonstrated [17]. In order to analyze the role of A. fumigatus siderophore production during growth initiation, we assessed the time course of conidial swelling and germ tube formation by *A. fumigatus wt, ΔsidA, ΔsidC, ΔsidD, ΔsidF,* and *ΔsidG* strains under iron-depleted conditions, iron-depleted conditions in the presence of BPS (blocking RIA), and under iron-replete conditions having either iron sulphate or TAFC as iron source (Tables 2 and 3) [7,28]. *wt* conidia were largely unaffected by the different conditions, as were those of *ΔsidD, ΔsidF,* and *ΔsidG* strains (unpublished data), indicating that, in the presence of sufficient intracellular siderophore, extracellular iron mobilization is not a requirement for efficient growth initiation under any of the conditions tested. In contrast, conidial swelling and germ tube generation by *ΔsidA* conidia were not observable until 4.7 and 6.3 h, respectively, after the *wt* in iron-depleted conditions, demonstrating that siderophore-mediated iron storage or utilization of intracellular iron is required for efficient growth initiation under iron limitation (Tables 2 and 3). *ΔsidA* swelling and germ tubes were completely absent under iron depletion in the presence of BPS, demonstrating that RIA can promote initiation of growth in the absence of siderophores, albeit at a considerably slower rate. An increase of extracellular iron availability partly overcame the *ΔsidA* defects. TAFC was marginally more effective for this than ferrous sulphate, indicating that a lack of HFC can be compensated by uptake of extracellular iron and that siderophore-mediated iron uptake can more readily support efficient germ tube formation than RIA in the absence of HFC. Consistently, therefore, *ΔsidC* conidia displayed a delay in both swelling and germ tube production during iron-depleted conditions, but to a lesser extent than that of *ΔsidA* conidia, emphasizing that the ability to synthesize extracellular siderophores supports efficient germ tube development in the absence of HFC.

**Table 2:**
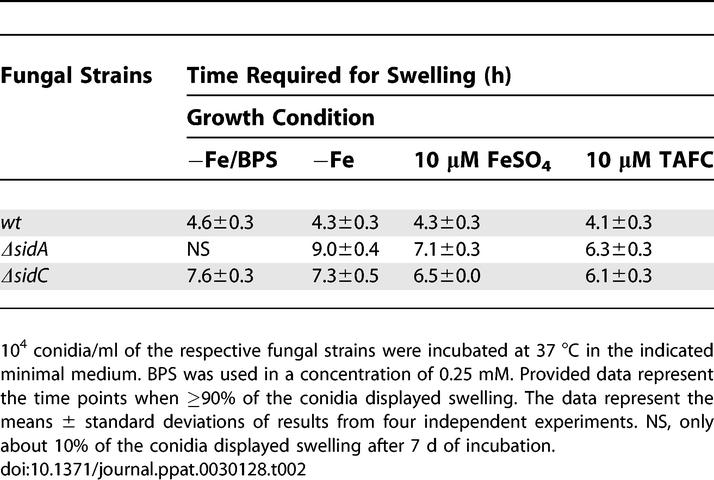
Conidial HFC Affects the Timing of Conidial Swelling

**Table 3:**
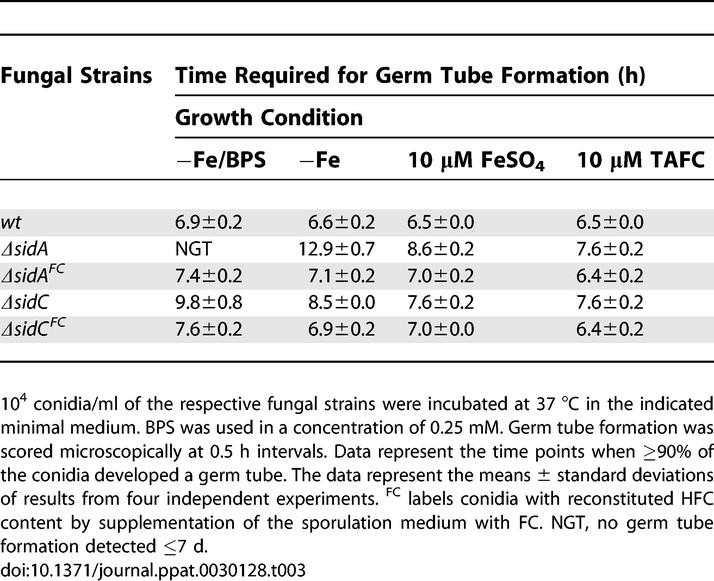
Conidial HFC Affects the Timing of Conidial Germ Tube Formation

As noted above, *ΔsidA* and *ΔsidC* conidia can be loaded with the HFC precursor FC, and such conidia approximated *wt* initiation of growth (Table 3), establishing that the described growth defects are indeed due to lack of HFC. Notably, FC supplementation (as the sole means of acquiring iron) permits a conidial HFC iron content sufficient to allow germ tube formation and elongation up to a length of 500 μm in vitro, as HFC-containing *ΔsidA* conidia stop growing at this stage in the presence of BPS (unpublished data).

### Impact of A. fumigatus Siderophores on Resistance to Iron Starvation and Oxidative Stress

**Figure 6:**
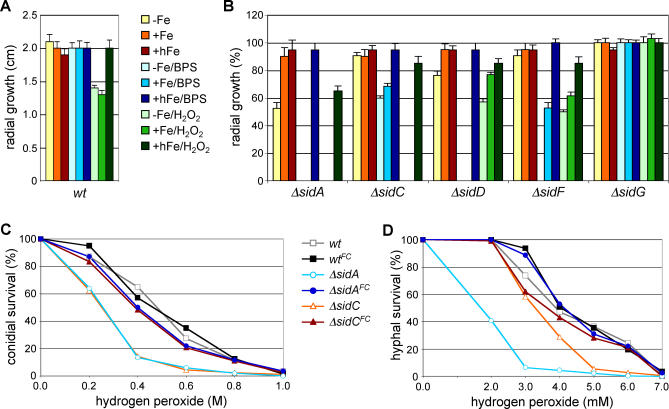
Impact of Extra- and Intracellular Siderophores on Resistance to Iron Limitation and Oxidative Stress (A) 10^4^ conidia of *wt* were point inoculated and radial growth was measured after 48 h at 37 °C on minimal medium lacking iron (−Fe), containing 10 μM FeSO_4_ (+Fe), 0.5 mM FeSO_4_ (hFe), 0.25 mM bathophenanthroline disulfonate (BPS), 2 mM H_2_O_2_, respectively. (B) Radial growth of respective mutant strains was determined as described in (A) and normalized to that of the *wt* grown in the same condition. The data in (A, B) represent the means ± standard deviations of results from three independent experiments. (C, D) Analysis of hydrogen peroxide sensitivity of conidia (C) and hyphae (D) was determined as described in Materials and Methods. The conidia used were harvested from plates containing 1.5 mM FeSO_4_ or 10 μM FC (^FC^). Samples were prepared in triplicate, and the standard deviation did not exceed 15%.

To analyze the role of extracellular and intracellular siderophores in resistance to iron starvation and to oxidative stress, radial growth rates of *ΔsidA, ΔsidC, ΔsidD, ΔsidF,* and *ΔsidG* were compared to that of *wt* under various stress-inducing growth conditions (Figure 6). In all assays performed (see below), *ΔsidG* behaved comparably to the *wt,* demonstrating that the TAFC precursor FSC, at least at the elevated levels observed in this mutant, can fully compensate an absence of TAFC. As shown previously [12], a complete lack of siderophores (*ΔsidA*) reduces the growth rate significantly during iron-depleted conditions. In comparison, absence of either the intracellular (*ΔsidC*) or the extracellular (*ΔsidD, ΔsidF*) siderophores under iron limitation caused a mild growth reduction, suggesting some redundancy in function for intra- and extracellular siderophores under these conditions. Interestingly, *ΔsidF* was less affected than *ΔsidD.* During iron-depleted conditions, inhibition of RIA by BPS completely impaired the growth of *ΔsidA, ΔsidD,* and *ΔsidF* and reduced the growth rate of *ΔsidC.* Compared to *ΔsidF, ΔsidA* and *ΔsidD* required a higher extracellular iron concentration to compensate the defect. Taken together, these data suggest that, in the absence of siderophore-mediated iron mobilization, RIA is an absolute requirement for surviving iron limitation, being able to compensate partially for a lack of intracellular or extracellular siderophores in vitro*.* Furthermore, intracellular siderophores are also required for promoting growth in the absence of RIA, although to a lesser extent compared to the extracellular siderophores because *ΔsidC* is less affected by BPS in iron-limiting conditions.

Detoxification of hydrogen (peroxide) depends on iron because catalases and peroxidases require heme as cofactor. Consistently, iron-depleted A. fumigatus is more sensitive to hydrogen peroxide than iron-replete cells (unpublished data). On the other hand, inappropriate iron storage can catalyze formation of reactive oxygen species. Deficiency of total (*ΔsidA*) and intracellular (*ΔsidC*) siderophores caused hypersensitivity to hydrogen peroxide during iron-depleted growth (Figure 6B). This defect was cured by an increase of extracellular iron availability, suggesting that the major role of intracellular siderophore is efficient iron utilization rather than iron detoxification. Deficiency in extracellular siderophores (*ΔsidD* and *ΔsidF*) rendered cells partially sensitive to hydrogen peroxide, and again, this defect was limited to iron-depleted conditions (Figure 6B).

To investigate the oxidative stress sensitivity of strains deficient in the extra- and/or intracellular siderophore in more detail, we analyzed conidial and hyphal killing by hydrogen peroxide in each mutant background. HFC-lacking conidia of both *ΔsidA* and *ΔsidC* showed increased sensitivity to hydrogen peroxide, and this defect was cured by HFC loading (Figure 6C). These data indicate that deficiency in conidial siderophore iron storage causes increased susceptibility to killing by hydrogen peroxide.

In a hyphal killing assay, *ΔsidA* was significantly more sensitive and *ΔsidC* slightly more sensitive than *wt* (Figure 6D), demonstrating that FC also plays a role in hyphal oxidative stress resistance. In contrast to *ΔsidA* and *ΔsidC,* conidia of *ΔsidD, ΔsidF,* and *ΔsidG* were as resistant as *wt* to hydrogen peroxide (unpublished data), suggesting that the increased sensitivity of *ΔsidD* and *ΔsidF,* found in the plate assays (Figure 6B), is due to sensitivity of hyphae only—probably due to iron deficiency caused by lack of extracellular siderophores.

To test the hypothesis that catalase deficiency underlies the observed oxidative stress sensitivity of *ΔsidA* and *ΔsidC,* we performed catalase staining on hyphal and conidial protein extracts. A. fumigatus produces three active catalases, Cat1 and Cat2 in hyphae, and CatA in conidia [29]. Mycelium of strains lacking both Cat1 and Cat2 exhibit only slightly increased sensitivity to hydrogen peroxide, whereas CatA deficiency results in significant increased sensitivity to hydrogen peroxide of conidia [29].

**Figure 7:**
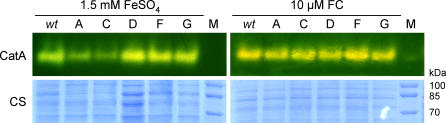
CatA Activity in *wt, ΔsidA*, *ΔsidC*, *ΔsidD*, *ΔsidF*, and *ΔsidG* A 40-μg conidial protein extract prepared from spores harvested from sporulation medium containing either 1.5 mM FeSO_4_ or 10 μM FC as iron source, respectively, was subject to native polyacrylamide gel electrophoresis (PAGE) and catalase (ferricyanide-negative) staining as described by Paris et al. [29]. As a control for loading and protein quality, the same samples were alternatively subject to SDS-PAGE (8%) and Coomassie staining (CS). Lane A, *ΔsidA;* lane C, *ΔsidC;* lane D, *ΔsidD;* lane F, *ΔsidF;* lane G, *ΔsidG.* M, molecular mass marker lane.

Catalase activity zymograms demonstrated that activity of hyphal Cat1 and Cat2 is decreased during iron-depleted compared to iron-replete conditions, but did not display any difference between *wt, ΔsidA, ΔsidC, ΔsidD, ΔsidF,* and *ΔsidG* strains (unpublished data). These data suggest that the increased oxidative stress sensitivity of the siderophore mutant strains during iron-depleted conditions is not due to decreased hyphal catalase activity. CatA activity was about the same in *wt, ΔsidD, ΔsidF,* and *ΔsidG* strains but significantly decreased in *ΔsidA* and *ΔsidC* conidia (Figure 7), which agrees with the increased hydrogen peroxide sensitivity of conidia from these strains. Supplementation of the sporulation medium with FC reconstituted CatA activity of *ΔsidA* and *ΔsidC* conidia, demonstrating that the catalase defect is caused by lack of conidial siderophore.

Taken together, all mutant impairments were most severe under iron-depleted conditions and at least partially reverted by an increase of extracellular iron availability, indicating that the observed defects are related to gene deletion–induced iron deficiency. Alternatively, or additionally, the defects might be related to accumulation of toxic intermediates, produced only during iron-depleted conditions. In this respect, the differing behaviour of *ΔsidD* and *ΔsidF* might indicate that abolition of TAFC synthesis at different steps of the biosynthetic pathway has varying consequences dependent upon different pathway intermediates interfering with metabolism.

### Both Intra- and Extracellular Siderophores Are Required for A. fumigatus Virulence

**Figure 8:**
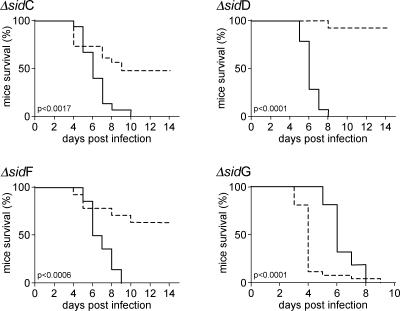
Analysis of Murine Survival following A. fumigatus Siderophore Mutant Infection Comparative survival of neutropenic mice following infection with *A. fumigatus ΔsidC, ΔsidD, ΔsidF,* and *ΔsidG* (broken lines) and corresponding complemented strains (solid lines). Mice were sacrificed when 20% of body weight with respect to the day of infection was reached.

**Figure 9:**
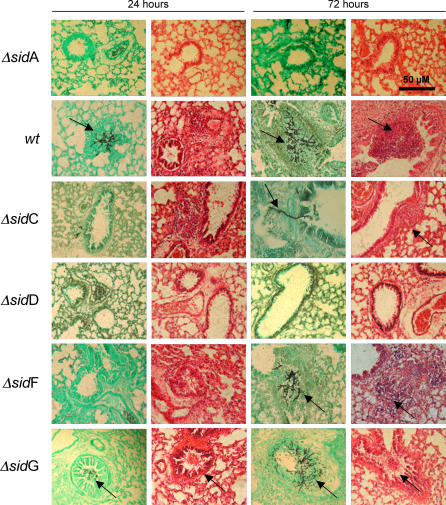
Histopathological Analysis of *Aspergillus*-Infected Murine Lung Sections Comparative histopathology of neutropenic murine lung sections following infection with *A. fumigatus wt* or *A. fumigatus ΔsidA, ΔsidC, ΔsidD, ΔsidF,* and *ΔsidG* mutants. Sections were sampled at 24 and 72 h post-infection, fixed in 4% ^v^/_v_ formaldehyde, and stained using Grocotts Methanamine Silver (GMS), or hematoxylin and eosin (HE). Infectious foci containing fungal hyphae and inflammatory lesions are indicated by arrows over GMS and HE sections, respectively.

To assess the relative contributions of intra- and extracellular siderophores to virulence of *A. fumigatus,* we compared the survival of neutropenic mice following infection with 5 × 10^5^ *A. fumigatus ΔsidA, ΔsidC, ΔsidD, ΔsidF,* or *ΔsidG* conidiospores to that of mice (*n* = 13) infected with an equivalent dose of the corresponding complemented strain (Figure 8). Following intranasal inoculation with a saline conidial suspension, mice were monitored for signs of respiratory distress and weighed daily. A cumulative weight loss of 20% body weight relative to that measured on the day of infection was taken as a stand-alone endpoint of experimentation. The median survival time of mice (*n* = 13) infected with the parental isolate ATCC46645 was 6 d, and 100% mortality was recorded for this group since no mouse infected with this *wt* isolate survived beyond the 11th day post-infection (Figure S2). Histopathological analysis of *wt*-infected lung tissue sections revealed numerous germinated spores and branching primary hyphae appearing as discreet pulmonary lesions in the bronchioles and alveoli at 24 h post-infection (Figure 9) and having an even distribution throughout the sections examined. Weight loss was steady from day +1 of the infection (unpublished data), and recruitment of inflammatory cells to foci of *wt A. fumigatus* infection was evident at 24 h, and substantial at 72 h, post-infection. This pathology stands in direct contrast to that of *ΔsidA*-infected mice, where virulence is completely attenuated [12] and neither germinated spores nor hyphal elements are observable at this infectious dose at similar time points of infection (Figure 9).

In comparison to survival following infection with a minimum of three independently gene-complemented strains (*n* = 15, Figure S3), survival of *ΔsidC*-infected mice (*n* = 23) was significantly increased (*p* = 0.0017, by log rank survival analysis), leading to 41% mortality and median survival time of 8 d among mice succumbing to infection (Figure 8). *ΔsidC* infection was characterized by a marked reduction in germinated spores at 24 h post-infection and a concomitant absence of inflammation relative to *wt* infection (Figure 9). Many phagocytosed conidia were evident at this time point (unpublished data), as well as extremely scarce incidences of germination and tissue invasion (Figure 9). At 72 h post-infection, hyphal moieties were detectable, but very scarce compared to the *wt*, and discreet inflammatory lesions were associated with such foci as evidenced by hematoxylin and eosin staining (Figure 9). Comparative infection with *ΔsidF* (*n* = 14) and corresponding complemented strains (*n* = 14, Figure S3) resulted in similarly attenuated virulence (*p* = 0.0006, by log rank survival analysis) resulting in 36% mortality and median survival of 6 d among mice succumbing to infection (Figure 8). Germinated spores were detectable by histopathological examination at the 24-h time point of infection; however, they were difficult to locate due to their sparse distribution within the sections examined. Mild inflammation was evident at this time point, as well as many instances of phagocytosed conidia (unpublished data). At 72 h post-infection, germinated spores and mycelial growths were evident with accompanying inflammatory cell recruitment (Figure 9). Once more, the frequency of such lesions is markedly reduced in comparison with the *wt* infection. *ΔsidD* infection (*n* = 13), however, was not fatal in neutropenic mice. With a single exception, mice survived the infectious challenge (Figure 8) and no significant weight loss was recorded despite renewed immunosuppression throughout the course of experimentation (unpublished data). *ΔsidD* is therefore severely (if not completely) attenuated for virulence in neutropenic mice (*p* < 0.0001 compared to *n* = 13 complemented strains). Viable conidia were not recovered from the lung of the mortally afflicted subject, though a degree of bacterial colonization of the lung was evident upon plating lung homogenates, and a severe drop in body weight commencing on day +4 was observed. We therefore reserve judgement on the cause of this particular fatality. Histopathology was comparable to that of *ΔsidA* infection, with a similar lack of germinating spores at any time point examined and no evidence of inflammatory response to the inoculum at the time points tested (Figure 8). Conversely, infection with *ΔsidG* was as virulent as the *wt* and demonstrated a similar pathology of infection (Figures 8 and 9).

**Figure 10:**
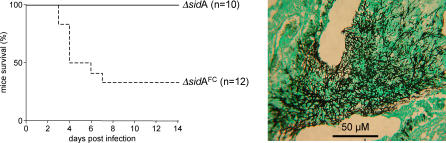
HFC-Mediated Rescue of *ΔsidA* Virulence in Neutropenic Mice Comparative survival of neutropenic mice (left panel) following infection with FC-supplemented (*ΔsidA^FC^*) and non-supplemented (*ΔsidA*) *ΔsidA* conidia, representing FC-loaded and unloaded conidia, respectively. Histopathological analysis (right panel) of Grocotts Methanamine Silver–stained tissue sections at 4 d post-infection reveals discreet mycelial lesions.

We previously demonstrated an absolute requirement for A. fumigatus siderophore biosynthesis for pathogenicity in neutropenic mammalian infections [12]. The analyses described here identify a clear requirement for both intra- and extracellular siderophores for full pathogenicity since abrogation of either category leads to attenuation of virulence. *ΔsidA* attenuation therefore likely results from the compound phenotype associated with total siderophore abrogation, including delayed germination, increased conidial and hyphal sensitivity to oxidative stress, and sub-optimal growth under iron-limiting conditions. To determine the contribution of germinative capacity and oxidative stress resistance to overall virulence phenotype, we exploited the capacity to load conidia artificially with HFC (through FC supplementation of the growth medium) and examined *ΔsidA* and *ΔsidC* virulence in the presence, and absence, of HFC. We reasoned that if the lack of siderophore-assisted germination and oxidative stress resistance was the sole basis of *ΔsidA* attenuation, then HFC-mediated rescue of *ΔsidA* germination in vivo would also completely rescue virulence. FC supplementation of *ΔsidA* growth for 5 d prior to infection partially restored pathogenicity, resulting in a 50% recovery of virulence (Figure 10). However, no increase in *ΔsidC* virulence was observed under similar experimental conditions (unpublished data). Complementation (see Materials and Methods) reversed all mutant virulence phenotypes (Figures 8 and S3), definitively linking attenuated phenotypes to inactivation of the respective gene.

## Discussion

The microbial quest for iron in mammalian hosts is crucial for successful pathogenesis as, in this environment, iron is tightly bound by carrier proteins such as transferrin, leaving free iron concentrations insufficient for sustenance of microbial growth. Most aerobic bacteria and fungi have genes encoding iron transport systems that become induced under iron limitation [30,31], among which siderophore-mediated iron transport provides a means of uptake, even for organisms which cannot themselves synthesize such molecules. Various bacteria produce extracellular siderophores, and in many cases their involvement in virulence has been demonstrated [30]. However, bacteria do not produce intracellular siderophores, but use ferritin and bacterioferritin for this purpose [32]. We have previously demonstrated [12] that in the aggressive, but poorly characterized, fungal pathogen *A. fumigatus,* a single genetic locus, *sidA,* directs biosynthesis of the extracellular siderophores FSC and TAFC (for mobilization of extracellular iron), and a hyphal siderophore FC (for hyphal iron storage). Coupled with a second high-affinity iron uptake mechanism, RIA, these low molecular mass ferric iron–specific chelators ensure a steady supply, and appropriate storage, of cellular iron. Abrogation of A. fumigatus siderophore biosynthesis by *sidA* deletion prevents initiation of mammalian infection, which cannot be supported by RIA alone. Consistently, inactivation of RIA by deletion of the high-affinity iron permease–encoding *ftrA* is inconsequential for virulence, identifying siderophore biosynthesis by this organism as paramount to successful pathogenesis. The absence of such biosynthetic pathways in mammals lends much promise to siderophore biosynthesis as the basis for therapy. To this end we have genetically delineated pathways of siderophore biosynthesis in A. fumigatus. Previously, the ornithine monooxygenase–encoding *sidA* was the only known gene, and its deletion, resulting in absence of all siderophore types, caused a compound phenotype comprising hypersensitivity to hydrogen peroxide, increased iron demand for germination, and reduced growth rate, in particular during iron-depleted conditions in vitro (this study and [12]). Here, we describe the identification and comparative mutational analysis of four novel A. fumigatus iron-regulated genes, *sidC, sidF, sidD,* and *sidG,* whose expression is repressed by iron, allowing elucidation of the A. fumigatus siderophore biosynthetic pathways downstream of *sidA* (Figure 1) and facilitating analysis of the relative contributions of extra- and intracellular siderophores in germ tube formation, sporulation, tolerance to iron depletion, oxidative stress resistance, and virulence.

Initial comparisons of siderophore production in mutant and *wt* backgrounds were highly informative with respect to ordering genetic loci within the expected biosynthetic pathways. The nonribosomal peptide synthetase SidC is required for biosynthesis of FC and HFC, whereas the acetyl transferase SidG, the acyltranferase SidF, and the nonribosomal peptide synthetase SidD are essential for biosynthesis of TAFC, suggesting the biosynthetic pathway shown in Figure 1. Analyses of respective siderophore biosynthetic mutants in comparison to the *wt* and *ΔsidA* isolates revealed a range of phenotypes attributable to siderophore deficiency. Moreover, these analyses identified a novel conidial siderophore in A. fumigatus, derived from FC by hydroxylation, which we termed hydroxyferricrocin. We found that similar to FC in A. nidulans [17], HFC is required for conidial iron storage in A. fumigatus (Table 1) and that its lack causes increased sensitivity to oxidative stress (Figure 6C), likely due, at least in part, to CatA deficiency (Figure 7), and delayed swelling and germ tube formation of conidia during iron depletion (Tables 2 and 3). Remarkably, therefore, the iron homeostatic machinery of A. fumigatus differs from that in the closely related, but negligibly virulent, model ascomycete A. nidulans in at least two aspects. A. fumigatus uses HFC as the conidial iron storage compound instead of FC and is able to assimilate iron reductively (RIA), which makes it more versatile with respect to iron acquisition [31].

The partial rescue of *ΔsidA* attenuation following reconstitution of the conidial HFC, which is possible by supplementation of the sporulation medium with the HFC precursor FC, in the absence of de novo synthesis of both extracellular and intracellular siderophores demonstrates the importance of the conidial siderophore during the initial phase of infection.

Prevention of HFC biosynthesis, as evidenced by measurement of conidial iron content in *ΔsidA* and *ΔsidC* backgrounds (Table 1), reduced conidial iron content by 67% compared to the *wt* isolate, and negated a role for extracellular siderophores in conidial iron storage since no difference in conidial iron content was discernable between *ΔsidA*, which lacks extracellular siderophores, and *ΔsidC*, which has a full complement of extracellular siderophores.

In fact, such comparative analyses of *ΔsidA* and *ΔsidC* provide a useful basis for determining the contribution of extracellular siderophores to several physiological processes. For example, while *ΔsidA* germination is significantly delayed or completely absent during iron deficiency whether in the presence or absence of RIA, *Δsid*C displays a far milder phenotype under both conditions, highlighting the importance of extracellular iron mobilization as a compensator for deficiency in intracellular iron storage and identifying an interdependency between different types of siderophore for certain cellular processes, such as germination. Other phenotypic manifestations of siderophore-mediated iron storage deficiency include growth retardation under iron limitation (regardless of RIA activity) and extreme sensitivity to oxidative stress, as measured by radial growth on solid agar, which was equally potent in both *ΔsidA* and *ΔsidC* backgrounds. The fact that *sidA* and *sidC* are equally required for full conidial resistance to oxidative stress but *ΔsidA* is avirulent and *ΔsidC* only partially attenuated demonstrates that this common feature cannot be the sole virulence determinant of these mutants, and indicates a crucial role also of extracellular siderophores, which are still produced by *ΔsidC*. Closer examination of the oxidative stress sensitivities revealed an important distinction between the two strains where, despite identical conidial sensitivities to hydrogen peroxide (Figure 6C), hyphal sensitivity to hydrogen peroxide was significantly greater for *ΔsidA* (Figure 6D), implicating extracellular siderophore production in hyphal tolerance to oxidative stress. Both mutants suffer catalase A deficiency (Figure 7), which correlates well with conidial sensitivity to hydrogen peroxide (Figure 6C). Distinguishable severity of phenotype in these two mutant backgrounds extends also to pathogenicity, where the partial attenuation of virulence observed following *ΔsidC* infection (Figure 8) presumably reflects the delayed germination, growth retardation, and oxidative stress phenotypes observed for this mutant in vitro. All observed defects were more pronounced under iron depletion, suggesting that the intracellular siderophore is required for optimal iron storage and possibly iron distribution rather than iron detoxification. Consequently, the increased sensitivities to oxidative stress in *ΔsidA* and *ΔsidC* backgrounds might be due to hampered detoxification that requires iron as cofactor; for example, the heme-containing catalases and peroxidases [4]. In light of the fact that catalase A deficiency alone has no role in virulence [29], resistance to oxidative stress in vivo, regardless of origin, must be supported by other as yet unidentified functions. An alternative explanation for *sidA*- and *sidC*-supported pathogenicity might be that disruption of iron homeostasis due to lack of the intracellular siderophore increases intrinsic oxidative stress. As found previously for A. nidulans [21], deficiency in the intracellular siderophore caused reduced production of spores, indicating a crucial role in conidiogenesis (Figure 5).

Considering similarly the effects of extracellular siderophore abrogation, certain physiological consequences are clearly unique to elimination of these compounds in A. fumigatus. In contrast to the iron deficiency–mediated growth retardation observed in the Δ*sidC* background, *ΔsidD* and *ΔsidF* mutants are completely incapable of growth in the absence of RIA when challenged with iron shortage. This highlights a dramatic shortfall in terms of compensatory mechanisms for extracellular siderophore deficiency, which is likely to extend to infection scenarios where the severe lack of available free iron would render RIA suboptimally effective. Partial sensitivity to oxidative stress is measurable by radial growth retardation for both *ΔsidD* and *ΔsidF* (Figure 6B) and both strains demonstrate attenuated virulence, although this phenotype is far more pronounced (or possibly absolute) for *ΔsidD*. By our analyses, the only basis for this difference lies in the amount of free iron supplementation required for each strain to rescue the effect of RIA inactivation, which is much higher for *ΔsidD* than *ΔsidF*. One might therefore hypothesize that RIA is able to support *ΔsidF* virulence to a greater extent than *ΔsidD* virulence. Alternatively, or additionally, the defects might be related to accumulation of intermediates of the blocked pathway, which is induced only during iron-depleted conditions, and interfere with metabolism. Notably, reversed-phase HPLC analysis combined with mass spectrometry indicated that the supernatant of iron-depleted *ΔsidD,* but not *ΔsidF*, contains elevated amounts of two compounds compared with *wt*. One of these could be identified as *N* ^5^-*cis*-anhydromevalonyl-*N* ^5^-hydroxyornithine, the direct precursor of FSC (unpublished data). In this respect, the increased sensitivity to iron depletion and oxidative stress of *ΔsidD*, compared to *ΔsidF*, suggests that blockage of TAFC synthesis at different steps of the biosynthetic pathway has different consequences. In accord with the in vitro phenotypes, deletion of *sidD* had a greater impact on virulence as compared to *sidF.* All *ΔsidD* and *ΔsidF* defects were compensated by an increase in extracellular iron availability, suggesting that these impairments are related to gene deletion–induced iron deficiency (Figure 6B). The attenuated virulence of *ΔsidD* and *ΔsidF* (Figure 8) clearly indicates induction of this pathway during infection. Notably, Cramer et al. [33] showed that *sidD* is the most highly expressed A. fumigatus NRPS-encoding gene following incubation with macrophages. Furthermore, we found significant induction of *sidC, sidD, sidF,* and *sidG* at the level of gene expression at an early stage of infection in neutropenic mice in genome-wide expression analyses (A. McDonagh, personal communication). Iron deficiency is unlikely to result from immunosuppressive regimen alone since many other microbes suffer iron stress–induced attenuation in immunocompetent murine models of infection, with the pulmonary pathogen Mycobacterium tuberculosis providing a good example [34].

During iron depletion, A. fumigatus usually excretes high amounts of TAFC and low amounts of the ultimate TAFC precursor FSC. Strains producing FSC in amounts comparable to those of TAFC produced by *wt*, but lacking TAFC due to deficiency in SidG, behave like *wt* under all conditions investigated, including virulence, demonstrating that FSC can satisfactorily replace TAFC as a siderophore in vitro and in vivo.

This study demonstrates the distinct roles of intra- and extracellular siderophores in iron homeostasis of A. fumigatus and reveals that the complete complement of intra- and extracellular siderophores is required for full virulence of this species. The predominant role for intracellular siderophores appears to lie with promoting germination and resisting oxidative stress, both of which require extracellular siderophores, whose absence or malfunction can be supported by RIA. The predominant role of extracellular siderophores, however, is to facilitate hyphal growth under iron limitation, particularly in circumstances where RIA is ineffective.

Recently, RIA was found to be dispensable for virulence of the plant pathogen Fusarium graminearum [35], whereas deficiency in the A. fumigatus SidD ortholog NPS6 caused loss of extracellular siderophores and reduction of virulence of *F. graminearum, Cochliobolus heterostrophus, C. miyabeanus,* and *Alternaria brassicicola* [23], demonstrating that siderophores are a common virulence determinant of at least some animal and plant pathogenic fungal species. Moreover, the intracellular siderophore has also been implicated in the virulence of Mangaporthe grisae in rice [36]. Nevertheless, the role of individual iron homeostasis-maintaining mechanisms in virulence largely depends on the pathogen–host system because the siderophores produced by the phytopathogenic basidiomycetes Ustilago maydis and Microbotryum violaceum do not contribute to their virulence [37,38], and because there are siderophore-lacking animal pathogenic ascomycetes, for example, *C. albicans,* and basidiomycetes, for example, *C. neoformans.* In C. albicans and *U. maydis,* RIA was found to be crucial for virulence [39,40]. Our analysis depicts complementary, but differential roles for distinct A. fumigatus siderophores, which appear to be employed for different purposes in vitro, and during infection, across a developmental spectrum. Under most circumstances, the combined abolishment of intra- and extracellular siderophore biosynthesis is required for extreme debilitation. An exception to this rule is posed by *sidD* inactivation, which matches *ΔsidA* in terms of virulence attenuation (Figure 8). Sharing absolute growth inhibition in the absence of RIA under iron depletion, the functions lacked by these mutants would seem to be equally attractive from a therapeutic standpoint.

## Materials and Methods

### Strains, growth conditions, and oligonucleotides.

Fungal strains (Table 4) were cultured at 37 °C in +Fe-*Aspergillus* minimal medium (AMM, iron-replete conditions) according to Pontecorvo et al. [41] containing 1% (wt/vol) glucose as carbon source, 20 mM glutamine as nitrogen source, and 10 μM FeSO_4_. For iron-depleted conditions, iron was omitted. For growth assays, the respective strains were point inoculated in a concentration of 10^4^ colony forming units (cfu)/ml on appropriately supplemented AMM plates and incubated for 48 h at 37 °C. In view of sporulation defects in siderophore mutant backgrounds, generation of spores from all strains for which phenotypic and virulence testing is described was performed on medium supplemented with 1.5 mM FeSO_4_, unless otherwise stated. For supplementation with FC or TAFC, the siderophores were used in the holo form (iron containing). Oligonucleotides are listed in Table S1.

**Table 4:**
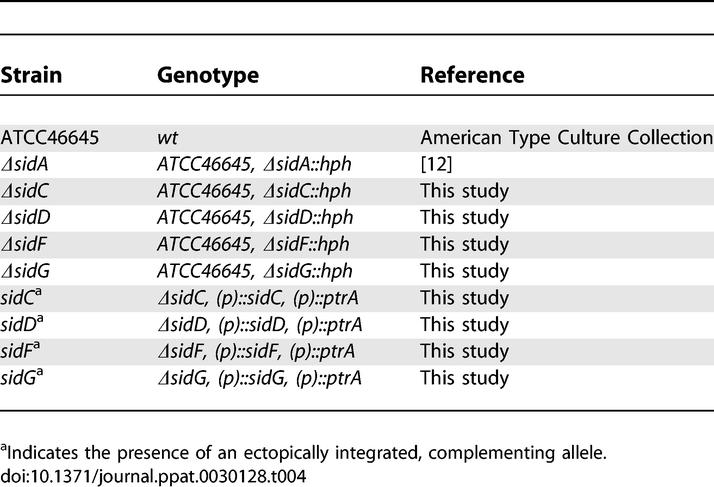
A. fumigatus Strains Used in This Study

### Northern analysis and DNA manipulations.

Total RNA was isolated using TRI reagent (Sigma-Aldrich, http://www.sigmaaldrich.com/) and northern analysis was performed according to Sambrook et al. [42]. Hybridization probes were generated by PCR using primer oAf_538_PS1-f and oAf_538_PS1-r for *sidD,* oAf_538_AT1-f and oAf_538_AT1-r for *sidF,* oAfAT1me and oAfAT2me for *sidG,* oAf_267_PS1-f and oAf_267_PS1-r for *sidC,* and oTubfum1 and oTUBFUMr for β-tubulin encoding *tubA*.

For extraction of genomic DNA, mycelia were homogenized, and DNA isolated according to Sambrook et al. [42]. E. coli DH5α strain was used as the host for general DNA propagations.

### Generation of A. fumigatus mutant strains.

For inactivation of *sidC, sidD, sidF,* and *sidG,* respective fragments including flanking regions were amplified from genomic DNA by PCR and subcloned into plasmid vectors. Genes were replaced by the hygromycin resistance marker gene *hph* [43] using standard techniques. Details are provided in Text S1. For transformation, deletion constructs were released from bacterial vectors and used for transformation of *wt* protoplasts. For the generation of *ΔsidF* and *ΔsidG* mutant strains, the bipartite marker technique was used [44]. Accurate gene deletion was confirmed by Southern hybridization. Mutant strains were complemented by co-transformation of respective plasmids (*sidF* and *sidG*) or cosmids (*sidC* and *sidD*) and plasmid pSK275 carrying the pyrithiamine resistance gene as a selection marker [45]. Direct selection of reconstitution alleles having single homologous insertions proved impossible despite several attempts, so we chose instead to complement each mutant strain by ectopic integration and test three independent complemented strains for each genetic defect generated. Single-copy ectopic integration in complemented mutant strains was verified by PCR and Southern hybridization.

### Identification, quantification, and purification of siderophores.

Characterization and quantification of siderophores was performed by reversed-phase HPLC chromatography according to Konetschny-Rapp et al. [46], as described by Oberegger et al. [15]. To analyze the conidial siderophore HFC, freeze-dried conidia were solubilized in a Mixer Mill 300 (Retsch, http://www.retsch.com/) and resuspended in 50 mM potassium phosphate buffer. After purification via an Amberlite XAD-16 resin (CWG, http://www.cwg.hu/) column, aliquots were analyzed by reversed-phase HPLC.

### Analysis of fungal damage by exogenous H_2_O_2_.

The sensitivity of conidia to killing by hydrogen peroxide was assayed as described by Han et al. [47]. Briefly, conidial suspensions approximating 10^5^ cfu/ml were incubated for 30 min at 20 °C with varying concentrations of hydrogen peroxide. To determine the number of surviving conidia, the spore suspensions were diluted 50-fold and plated on +Fe-AMM. Following incubation for 24 h at 37 °C, colonies were counted and normalized to that without hydrogen peroxide treatment.

The sensitivity of hyphae to hydrogen peroxide was estimated by using a modification of the protocol of Kawasaki et al. [48]. Approximately 150 conidia were plated on iron-replete minimal medium and grown at 37 °C for 19 h—at this time point small colonies were countable. Subsequently, the plates were overlaid with 4.5 ml of the same medium as top-agar but containing the indicated concentration of hydrogen peroxide. After further incubation for 24 h at 37 °C, colonies able to resume growth were counted as survivors and normalized to the number before hydrogen peroxide treatment.

### Murine infections.

Murine infections were performed under UK Home Office Project Licence PPL/70/5361 in dedicated facilities at Imperial College London. Outbred male mice (strain CD1, 18–22 g; Harlan Ortech, http://www.harlan.com/) were housed in individually vented cages. Mice were immunosuppressed as previously described [12]. A. fumigatus spores for inoculations were grown on *Aspergillus* complete medium, containing 5 mM ammonium (+)-tartrate, 200 mM NaH_2_PO_4_, and 1.5 mM FeSO_4_ for 5 d prior to infection. Conidia were freshly harvested using sterile saline (Baxter Healthcare, http://www.baxterhealthcare.co.uk/) and filtered through Miracloth (Calbiochem, http://www.emdbiosciences.com/g.asp?f=CBC/home.html). Conidial suspensions were spun for 5 min at 3,000*g,* washed twice with sterile saline, counted using a hemocytometer, and resuspended at a concentration of 6.25 × 10^6^–1.25 × 10^7^ cfu/ml. Viable counts from administered inocula were determined following serial dilution by plating on *Aspergillus* complete medium containing 5 mM ammonium (+)-tartrate, 200 mM NaH_2_PO_4_, and 1.5 mM FeSO_4_ and growth at 37 °C. Mice were anesthetized by halothane inhalation and infected by intranasal instillation of 2.5 × 10^5^–5 × 10^5^ conidia in 40 μl of saline. Mice were weighed every 24 h from day 3 and visual inspections made twice daily. In the majority of cases the end point for survival experimentation was a 20% reduction in body weight measured from the day of infection, at which point mice were sacrificed. Lungs for histological sectioning were removed immediately after sacrifice and fixed in 4% ^v^/_v_ formaldehyde (Sigma). Lungs were embedded in paraffin prior to sectioning and stained with hemotoxylin and eosin or light green and Grocott's Methenamine Silver.

## Supporting Information

Figure S1 Growth and Siderophore Status of A. fumigatus Gene-Complemented Siderophore Mutants Radial growth was determined as described in Figure 5 and normalized to that of the *wt* (A). Production of TAFC (B) and FC (C) was determined as described in Figure 3 and normalized to those of the *wt.*
(571 KB TIF)

Figure S2 Survival of Neutropenic Mice following Infection with A. fumigatus ATCC46445 (*n* = 13) Survival of neutropenic CD1 mice following intranasal infection with 2 × 10^5^ conidia of the wild-type isolate ATCC46645, which provides the gentic background for all strains constructed in this study.
(68 KB TIF)

Figure S3 Survival (Days) of Neutropenic Mice following Infection with Gene-Complemented Siderophore-Deficient A. fumigatus Strains Survival of gene-complemented strains is indicated by day of sacrifice for every strain tested.
(158 KB TIF)

Table S1 Primers Used in This Study (57 KB DOC)

Text S1 Detailed Description of Mutant Strain Construction (46 KB DOC)

### Accession Numbers

The A. fumigatus GenBank/NCBI (http://www.ncbi.nlm.nih.gov/) nucleotide and amino acid sequences, respectively, for the genetic loci described in this publication are *sidC* (XM_747995 and XP_753088.1), *sidD* (XM_743569 and XP_748662.1), *sidF* (XM_743567 and XP_748660.1), and *sidG* (XM_743592 and XP_748685.1).

## Abbreviations

BPS: bathophenanthroline disulfonate
FC: ferricrocin
FSC: fusarinine C
HFC: hydroxyferricrocin
HPLC: high-performance liquid chromatography
RIA: reductive iron assimilation
TAFC: triacetylfusarinine C
*wt*: wild type
