# Supplementary material for: Distinct Roles for Intra- and Extracellular Siderophores during Aspergillus fumigatus Infection
Source: PLoS Pathog. 2007 Sep 7;3(9):e128. doi: 10.1371/journal.ppat.0030128 (PMC1971116; doi:10.1371/journal.ppat.0030128)
Supplement: Supplementary file 1 [file ppat.0030128.sd001.doc]

**Text S1: Detailed description of mutant strain construction**

To enable inactivation of *sidC*, a 4.9-kb fragment of genomic DNA containing *A. fumigatus sidC* was amplified using primers oAF_267_KoPS1-f and oAF_267_KoPS1-r and cloned into pGEM-T (Promega). The resulting plasmid was sequenced and termed pSIDC. A 1.8-kb *Bgl*II-*Hind*III fragment of pSIDC was replaced with a 4.0-kb fragment from pAN7-1 [43] containing the hygromycin B (*hph*) selection marker. The resultant 7.1-kb *Sma*I fragment was gel purified and used to transform *A. fumigatus* ATCC46645. This results in a *sidC*-null allele, removing amino acids 2836 – 3423 of *sidC*.

To generate a *ΔsidD* allele, a 5.1-kb fragment was amplified using primers oAF_538_KoPS1-f and oAF_538_KoPS1-r, subsequently subcloned into pGEM-T, sequenced and termed pSIDD. A 3.9-kb *Bgl*II*-XbaI* fragment of vector pAN7-1 containing *hph*, replaced an internal 2.4-kb *BglII-XbaI* fragment. For transformation of ATCC46645, the gel–purified 6.5-kb *Nar*I fragment was used. In the generated mutant allele of *ΔsidD-hph* the deleted section encompasses the region encoding amino acids 305 - 1120 of *sidD*.

For generating *ΔsidF* and *ΔsidG* mutant strains the bipartite marker technique was used [44]. Briefly, *A. fumigatus* strain ATCC46645 was co-transformed with two DNA constructs, each containing an incomplete fragment of *hph* fused to 1.2-kb, and 1.4-kb of *sidF* flanking sequences, respectively. These marker fragments shared a 447-bp overlap within the *hph* cassette, which served as a potential recombination site during transformation. During transformation, homologous integration of each fragment into the genome flanking *sidF* allows recombination of the *hph* fragments and generation of the intact resistance gene at the site of recombination. Two rounds of PCR generated each fragment. First, each flanking region was amplified from ATCC46645 genomic DNA using primer oAT1-1 and oAT1-2 for flanking region A (1.2 kb), and oAT1-3 and oAT1-4 for flanking region B (1.4 kb). Subsequent to gel-purification, the fragments were digested with *Sac*I and *Xba*I, respectively. The *hph* selection marker was released from plasmid pAN7-1 by digestion with *Sac*I and *Xba*I, and ligated with the two flanking regions A and B described above. For generation of *Δsid*F*,* two overlapping fragments were amplified from the ligation product using primers oAT1-5 and ohph14 for fragment C (2.7-kb) and primers oAT1-6 and ohph15 for fragment D (2.8-kb). Subsequently ATCC46645 was transformed simultaneously with the overlapping fragments C and D. In the generated mutant allele of *ΔsidF-hph* the deleted region encodes for amino acids 5 - 451 of *sidF*.

To construct a *sidG* deletion mutant, the two fragments flanking the deleted region of *sidG* were amplified by PCR using the primers oAT2-1 and oAT2-4 for flanking region A (1.7 kb), oAT2-2 and oAT2-3 for flanking region B (1.3 kb). Subsequent to gel-purification, the fragment A was digested with *Xho*I and fragment B with *Hin*dIII, respectively. The *hph* selection marker was released from pAN7-1 via digest with *Xho*I and *Hind*III, and ligated with the two flanking regions A and B. For generation of *ΔsidG*, two overlapping fragments were amplified from the ligation product using primers oAT2-5 and ohph14 for fragment C (3.5-kb) and primers oAT2-6 and ohph15 for fragment D (2.3-kb). The fragments C and D were used together to transform *A. fumigatus* ATCC46645. In the *ΔsidG* mutant allele, *hph* replaced the complete coding region plus 511 nt upstream and 118 nt downstream of *sidG*.

To complement the extraordinarily large genes *sidD* and *sidC* an *A. fumigatus* cosmid library was used. Cosmids containing the gene to be recovered were identified by colony filter hybridization using gene-specific probes. Subsequently, the *sidC* containing cosmide psidC-COS was used to rescue *ΔsidC* and to complement *sidD* a fragment was released from psidD-COS via restriction with *Bam*HI and *Nhe*I.

*sidF* was found to be located adjacent to *sidD*. A *sidF*-containing fragment was released from psidD-COS via digestion with *Hind*III and *Eco*RV and used to complement the mutant allele of *sidF*. In all described complementation pyrithiamine encoded on the plasmid pSK275 was used as a selection marker [45].

To complement *ΔsidG* a PCR fragment containing the *sidG* locus including promoter and terminator was co-transformed with pSK275. The fragment was amplified using primers oAT2-5 and oAT2-6 and gel-purified prior to transformation. Gene disruption at the correct locus was confirmed by Southern hybridization. Single copy ectopic integration in all complemented mutant strains was verified by PCR and Southern hybridization.
