# Supplementary material for: Distinct Roles for Intra- and Extracellular Siderophores during Aspergillus fumigatus Infection
Source: PLoS Pathog. 2007 Sep 7;3(9):e128. doi: 10.1371/journal.ppat.0030128 (PMC1971116; doi:10.1371/journal.ppat.0030128)
Supplement: Supplementary file 2 [file ppat.0030128.st001.doc]

**Table S1. Primers used in this study**

| Primer | Sequence 5' - 3' | * |
| --- | --- | --- |
| oAF_267_KoPS1-f | CCGTTGTGCCAGTGCCATTTG |  |
| oAF_267_KoPS1-r | GGCCCCGGGAACTGGTAATGAGCGTC | *Sma*I* |
| oAf_267_PS1-f | CGGCATCGAGAAGCAAAG |  |
| oAf_267_PS1-r | CGGATCTTGGTTTGGTCG |  |
| oAf_538_PS1-f | TTGGCGAGAGGAGAGATG |  |
| oAf_538_PS1-r | TACGATGGGTGGTCAGAG |  |
| oAF_538_KoPS1-f | GGAGGCGCCGTTGTTTCCCTCGAC | *Nar*I* |
| oAF_538_KoPS1-r | TTTCCGCAGATGTATCGAGTC |  |
| oAf_538_AT1-f | CCTCATCCCTATCTCACC |  |
| oAf_538_AT1-r | AGTTTTGAGCGAGAGGGG |  |
| oAT1-1 | GCAGATCGATAACTTAGACGGCCTCCA | *Cla*I* |
| oAT1-2 | CTCGGAGCTCCTTTGAGTCGCCATCGC | *Sac*I* |
| oAT1-3 | CTGGAATCTAGAGATCGGATGGCGTGGG | *Xba*I* |
| oAT1-4 | CTGCAAGCTTATGGGGTTGGCACTAAGC | *Hind*III* |
| oAT1-5 | AATGCTCGTACTCCCTCG |  |
| oAT1-6 | GGCTTGGCCTAATACCTG |  |
| oAfAT1me | ACAATCAAGGCTCAGCCC |  |
| oAfAT2me | ACTTCGAGTCATGCTGGG |  |
| oAT2-1 | AAGGATCGATGGAATATGACGAACCCGC | *Cla*I* |
| oAT2-2 | GATATTTTAAATACCTCATGGCGTGCAAC | *Dra*I* |
| oAT2-3 | GTGTGCGGCCGCGTGTACCTCTTGCTTCCC | *Not*I* |
| oAT2-4 | ACTCTCGAGGCATCACCCAACATCCTC | *Xho*I* |
| oAT2-5 | GCCCACCAAACTGTCTTC |  |
| oAT2-6 | GCGTATGGAGCCAAGAGA |  |
| oTubfum1 | ATATGTTCCTCGTGCCGTTC |  |
| oTUBFUMr | CCTTACCACGGAAAATGGCA |  |
| ohph14 | GAAGATGTTGGCGACCTC |  |
| ohph15 | GAGAGCCTGACCTATTGC |  |

*indicates the add-on restriction enzyme site (underlined).
